# Evaluation of Functional Lower Urinary Tract Dysfunction in Children: Are the Physicians Complying with the Current Guidelines?

**DOI:** 10.1155/2013/341606

**Published:** 2013-04-23

**Authors:** Mesrur Selcuk Silay, Ahmet Ruknettin Aslan, Erim Erdem, Zafer Tandogdu, Serdar Tekgul

**Affiliations:** ^1^Department of Urology, Faculty of Medicine, Bezmialem Vakif University, Adnan Menderes Bulvarı, Fatih 34093, Istanbul, Turkey; ^2^Department of Urology, Haydarpasa Numune Training and Research Hospital, Tıbbiye Caddesi, No. 40, Istanbul, Turkey; ^3^Department of Urology, Faculty of Medicine, Mersin University, 33343 Mersin, Turkey; ^4^Department of Urology, Mardin State Hospital, Vali Ozan Caddesi, Mardin Merkez, Mardin, Turkey; ^5^Department of Urology, Faculty of Medicine, Hacettepe University, Sıhhiye, 06100 Ankara, Turkey

## Abstract

*Objective*. To elucidate whether the diagnostic and treatment approaches of the physicians for functional lower urinary tract dysfunction (LUTD) in children is complying with the current guidelines. *Material and Methods*. We have conducted an internet-based national survey for the physicians from different departments randomly sampled from the database of Turkish Paediatric Urology Society. Participants were asked to answer two-page questionnaire consisting of 4 main sections: “demography,” “working conditions,” “daily practice,” and “scientific knowledge.” Kruskal Wallis and multiple logistic regression were used for statistical analyses. *Results*. Of the 117 departments a total of 93 have completed the survey (*n*: 58 urology; *n*: 35 paediatric nephrology). Routine use of a questionnaire with validated symptom scoring system was found to be 13.9%. Of the participants, only 38.7% were asking all of the patients to fill the bladder diary. During treatment, only 24.7% were applying standard urotherapy for every patient. Almost half of the clinicians (45.1%) believed that they were personally insufficient during the evaluation of those children. Finally, 86% reported that children with LUTD were not adequately approached. *Conclusions*. Evaluation of LUTD in children is not complying with the current guidelines. General approach for those children needs to be revisited by the clinicians.

## 1. Introduction

Functional lower urinary tract (LUT) dysfunction without an overt uropathy and neuropathy is a highly prevalent disease among children. Although the incidence is reported to be as high as 21.8% in school-age, the evaluation of those children is usually underestimated [1]. A spectrum of nonneurogenic voiding disorders starting from giggle incontinence towards the Hinman syndrome may cause LUT symptoms [2]. Additionally, it is clearly associated with urinary tract infections (UTI), vesicoureteral reflux, and psychological disorders [3]. Therefore, the accurate investigation of those children is utmost important for the true diagnosis and the treatment. 

International Children's Continence Society (ICCS) pointed out that there is a confusion in the definitions between different disciplines, and they have previously standardized the terminology of LUT functions in children [4]. In that study, the storage and voiding symptoms are defined, and the tools of investigation for the assessment of LUT in childhood are established. Both in the ICCS study and the European Urology Guidelines of pediatric urology, a stepwise approach has been recommended during the diagnosis and the treatment [4, 5]. In this study, we aimed to elucidate whether the clinical approaches and the perceptions of the physicians for LUT dysfunction in children are complying with the current guidelines.

## 2. Materials and Methods

This study was organized by the “Voiding Dysfunction Study Group” of the Turkish Paediatric Urology Society. We have conducted an internet-based multicenter national survey for urology residents and pediatric nephrologists from the database of Turkish Paediatric Urology Society. A questionnaire regarding the voiding dysfunction in children was designed to assess the current level of understanding, therapeutic approach, and the adequacy of different departments and disciplines. The questions were prepared in a multiple-choice fashion. Participants were asked to answer a two-page questionnaire including 20 questions taking 3 minutes to complete. One physician from each department is randomly sampled, and the survey was delivered accordingly. A second reminder e-mail was sent to nonresponders in two weeks. If there still was no response, an e-mail to another physician from the same department was sent two weeks later. 

Questionnaire was consisting of 4 main sections: “demography,” “working conditions,” “daily practice,” and “scientific knowledge.” In the first part of the survey, the participants were asked to answer the demographic questions including current academic position, location, and the type of the hospital they work in. The second part for “working conditions” included the outpatient clinical conditions, the available urodynamic equipments, and the burden of the pediatric patients they examine in a month. The third part related to the daily practice included the use of the bladder diary, a questionnaire with a validated symptom scoring system, the application of the standard urotherapy, and the time they spent during the first visit. Finally, the last part was about testing the scientific knowledge including five basic questions. 

After an overall two months of reply period, the results were collected into a computer-based system and the ratios were automatically calculated. In some of the questions, a comparison between the answers of the urologists and paediatric nephrologists was performed. For this purpose, Kruskal Wallis test and multiple logistic regression were used for statistical analyses. The values were provided as mean ± standard deviation of the mean (SD). The study was approved by the local ethical committee and complies with Helsinki declaration. 

## 3. Results

Of the 117 departments, a total of 93 clinicians from different departments have completed the survey (*n*: 58 urology; *n*: 35 paediatric nephrology). The total response rate was 79.4%. Of the physicians, 61 (65.5%) were working at the university hospitals whereas the rest of them were at the teaching hospitals. Pediatric urology outpatient was separate from the adult outpatient in almost half of the urology departments (*n* = 28, 48.2%). The majority of the participants (64.5%, *n* = 60) revealed that children with LUTD were constituting 20% of their paediatric outpatients. The availability of the urodynamic equipments in the departments was as follows: uroflowmetry: 87% (*n* = 81), invasive urodynamics (cystometrography + pressure-flow studies): 65.5% (*n* = 61), electromyography (EMG): 43% (*n* = 40), and videourodynamics: 47.3% (*n* = 44). 

**Figure 1:**
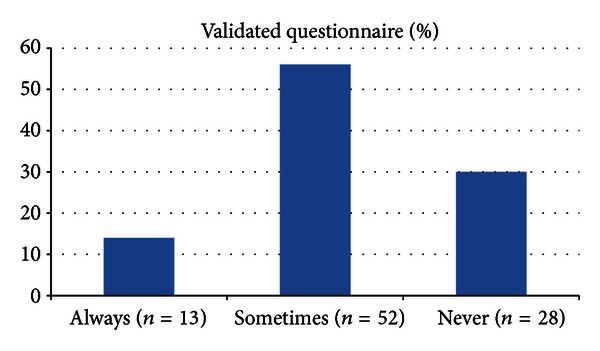
Application rates of the questionnaire with validated symptom scoring system.

**Figure 2:**
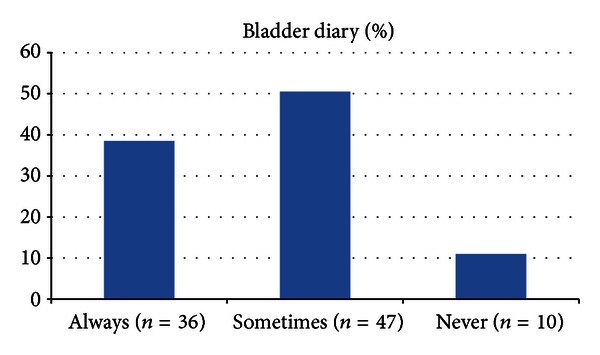
Application rates of the bladder diary.

**Figure 3:**
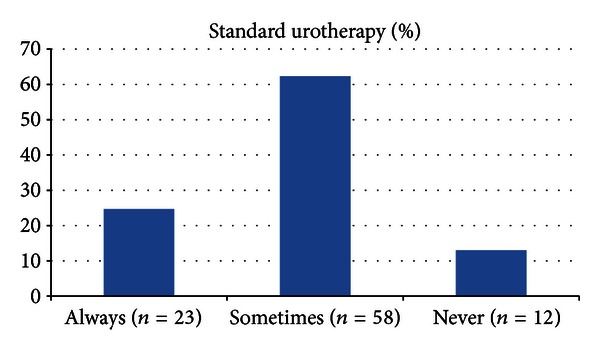
Application rates of the standard urotherapy.

The time spent by the physician during the first office visit of the children with LUTD was more than 10 minutes in only 37.6% (*n* = 35). The use of a validated questionnaire with symptom scoring system is summarized in the Figure 1. The application rates of the bladder diary and standard urotherapy are summarized in Figures 2 and 3, respectively. Of the respondents, 68.8% (*n* = 64) truly answered the 5 basic questions related to LUTD in children. 

The question assessing the self-sufficiency of the physicians revealed that only 38.7% (*n* = 36) believed they were competent in the evaluation of children with LUTD. Fifteen of the respondents (16.2%) felt moderately adequate and 42 (45.1%) reported they were personally insufficient. Finally, 86% (*n* = 80) of the participants reported that children with LUTD were not adequately approached. The reasons for the insufficient evaluation of those children were as follows: lack of time (*n* = 63, 67.5%), complexity of the disease (*n* = 20, 21.5%), and lack of knowledge (*n* = 10, 10.5%). The statistical comparison of some of the previous parameters between urology and pediatric nephrology departments is given in Table 1.

## 4. Discussion

Our results provide that the general approach for children having LUTS is seriously lacking. When taking history, although a structured approach is strongly recommended, application of a validated questionnaire and the bladder diary is clearly underestimated. Additionally, although standard urotherapy is the starting point in the treatment of those children, only 1/4 of the physicians seem like obeying the current guidelines. Finally, the self-assurance of the physicians in the evaluation of the children with LUTD is critically insufficient.

LUTD and daytime lower urinary tract conditions are very common among children. The symptoms are manifestations of variety of disorders, and their management can be perplexing due to the complexity of the terminologies used [6]. Therefore, the ICCS has previously reported the standardization study in order to prevent the clinicians from this semantic confusion [4]. Additionally, the European urology guidelines on pediatric urology recommend a structured approach for LUTD in children and also have been a useful reference for the clinicians [5]. However, due to the complexity of the disease, there are still some gaps in the diagnosis and the treatment strategies among the physicians. In our study, we have tried to elucidate this point and evaluate whether the general approaches of urologists and paediatric nephrologists are complying with the current guidelines. 

During the diagnosis of LUTD, a noninvasive stepwise approach is recommended. Detailed history, examination, uroflowmetry, ultrasound, and voiding diary are essential for a structured approach. Therefore, it is highly recommended that using a questionnaire as a checklist with a validated symptom scoring system is very beneficial [7, 8]. It has been stated that such an objective evaluation will not only allow diagnosis but also will provide monitoring the response to treatment. Hence, in our study, we have asked the participants whether they are using this diagnostic tool in their daily practice. Unfortunately, we have found that the majority of the physicians (86%, *n* = 80) are not routinely using a questionnaire when evaluating those children. We believe that this is one of the leading factors for the insufficient evaluation of the children with LUTD. 

Second, application of a bladder diary is not only a part of the diagnosis but also a part of the treatment. One of the components of the standard urotherapy is the demonstration of the voiding characteristics of the child by using a bladder diary or frequency-volume charts. It has been reported that voiding diary is mandatory to determine the child's voiding and drinking habits [5]. Therefore, in our study, we have asked the participants how frequent are they asking the patients to fill the bladder diary. Again, we have found that the routine application rate was only 38.7% among all physicians. We believe that this should be another important point that needs to be raised between the clinicians and enhancing the awareness is a must. 

Standard urotherapy is defined as the nonsurgical and nonpharmacological treatment of functional LUTD in children [5]. The main objective of urotherapy is decreasing the voiding symptoms via the rehabilitation of the bladder. There are several noninvasive components of this therapy:

1. to inform the patient and the parents about the normal urinary function and show them how their child deviates from the normal;
2. giving advices about the lifestyle, voiding, and drinking habits;
3. using a bladder diary;
4. supporting the families by regular followup.

They constitute the basis of the standard urotherapy. It has been demonstrated that solely application of the standard urotherapy is resulting with an improvement almost in 80% of the children [9–14]. Therefore, regardless of the type of the LUTD, this bladder training programme is considered to be mandatory before starting any specific interventions. From this perspective, we have investigated the application rate of urotherapy between the clinicians. Unfortunately, we have found that only 24.7% were routinely applying standard urotherapy for their patients. 

When taken all together, the very low application rates of questionnaire with validated symptom scoring system, bladder diary, and standard urotherapy, it is very clear why those children with LUTD are not appropriately evaluated. We have demonstrated that the majority of the respondents (86%) were thinking that those children were not adequately approached. The predominant reason for this comment was the lack of time (67.5%) during the busy daily schedule. This result might have also affected the self-sufficiency of the physicians, and only about one-third of them were feeling competent in the treatment of children with LUTD. We believe that insufficient evaluation, treatment, and the self-assurance are all linked with each other and this seems to be a womb-to-tomb problem. In our opinion, these factors should be the most important tasks of the professional societies in the field of paediatric urology in order to increase the awareness among the physicians. 

One might say that one of the limitations of our study is the relatively low number of participants. However, in this study, only one participant from each department is targeted and the survey was delivered accordingly. If no response was gathered, then another e-mail to a physician from the same department was sent. Finally, we could be able to get response from 93/117 departments in all over the country. Hence, we had the opportunity to include the majority of the departments and enhanced the diversity. However, we agree that our study would be superior if the other physicians from the other countries could be included into the study. 

## 5. Conclusions

Our results clearly demonstrate that evaluation of children with LUTD is seriously lacking and not complying with the current guidelines. We have shown that there are serious defects both during the diagnosis and the treatment. The self-assurance of the physicians was also found to be very low. General approach for those children needs to be revised by the clinicians. 

## Figures and Tables

**Table 1:** Comparison of the parameters between urology and paediatric nephrology departments.

Parameters	Urology	P. nephrology	*P* value
Available equipments:			
Uroflowmetry	56 (96.5)	25 (71.4)	0.001*
Invasive urodynamics	47 (81.0)	14 (40.0)	0.001*
EMG	28 (50.0)	12 (34.2)	0.1*
Videourodynamics	26 (44.8)	18 (51.4)	0.3*
Routine use of questionnaire	7 (12.0)	9 (25.7)	>0.13°
Routine use of bladder diary	24 (41.3)	12 (34.2)	>0.56°
Routine application of urotherapy	16 (27.5)	7 (20.0)	<0.34°
Time spent during first visit (min)			
<10′	41 (70.7)	16 (45.7)	<0.02°
>10′	17 (29.3)	19 (54.3)	

*Kruskall Wallis test.

°Multiple logistic regression analyses.
